# 岩藻糖基化与肿瘤

**DOI:** 10.3779/j.issn.1009-3419.2016.11.07

**Published:** 2016-11-20

**Affiliations:** 100071 北京，中国人民解放军军事医学科学院附属医院肺部肿瘤科 Department of Lung Oncology, Affiliated Hospital of the PLA Military Academy of Medical Sciences, Beijing 100071, China

**Keywords:** 岩藻糖基化, 肿瘤, 治疗, Fucosylation, Neoplasms, Therapy

## Abstract

岩藻糖基化是重要的糖基化修饰方式，在哺乳动物中发挥重要作用，其参与ABO血型H抗原、Lewis血型抗原形成、选择素介导的白细胞外渗或归巢、宿主病原相互作用及信号通路修饰。在多种肿瘤中存在岩藻糖基化异常，其在肿瘤生长、侵袭、转移、免疫逃逸以及药物敏感性方面发挥重要作用，与肺癌的发生发展及预后密切相关。因此，靶向肿瘤中异常岩藻糖基化可能成为治疗肿瘤的新策略。本文将对岩藻糖基化在肿瘤发生发展中的作用进行综述。

## Abstract (en)

Fucosylation is one of the most important glycosylation in mamal, involving biosynthesis of blood H antigen and Lewis antigen, selectin mediated intravasion and homing of leukocyte, host-microbe interactions, modification of signaling. Alterations in the expression of fucosylated oligosaccharides have been observed in several cancer, playing a role in proliferation, invasion, metastasis, immune escape and drug-sensitiity, especially in lung cancer. Targeting the abnormality of fucosylaion in cancer will become a new strategy. The role of fucosylation in cancer will be reviewed in this article.

50%以上的真核生物蛋白存在翻译后糖基化修饰，影响蛋白质折叠、稳定、运输、分泌以及生物功能^[1-3]^。在哺乳动物寡糖合成系统中，岩藻糖是十种单糖之一，岩藻糖基化是糖蛋白、糖脂寡糖修饰中最常见的修饰方式；已知的岩藻糖修饰有ABO血型系统的H抗原、Lewis血型抗原；岩藻糖还参与选择素介导的白细胞外渗或淋巴细胞归巢、病原宿主相互作用、信号转导通路的修饰；在多种病理情况下（如免疫、肿瘤），存在岩藻糖基化水平异常^[4]^。白细胞粘附缺陷Ⅱ患者岩藻糖基化异常导致白细胞与内皮细胞粘附缺陷^[5]^。Baumann等^[6]^于1979年首次报道了肝癌细胞中的岩藻糖基化的存在。AFP-L3，岩藻糖基化的甲胎蛋白（α-fetoprotein, AFP），AFP三种亚型之一，在日本（1996年）和美国（2005年）批准作为肝细胞肝癌的肿瘤标志物^[7, 8]^。随着蛋白质组学以及糖生物学研究手段的进步，肿瘤中越来越多的异常岩藻糖基化被发现，其在肿瘤细胞生长、侵袭、转移、免疫逃逸以及治疗抵抗方面发挥重要作用。

## 1 肿瘤中异常岩藻糖基化

### 1.1 生理条件下岩藻糖基化过程

岩藻糖基化是典型的末端修饰方式。包括N聚糖、粘蛋白型O糖以及直接与蛋白质丝/苏氨酸残基羟基上相连的岩藻糖^[4]^。GDP岩藻糖是岩藻糖基化的唯一供体，其在细胞质中合成，而后通过GDP岩藻糖转运体（GDP-L-fucose transporter, GDP-L-Fuc Tr）进入内质网或高尔基体^[9, 10]^。哺乳动物可通过两种途径合成GDP岩藻糖。从头合成途径：通过3步反应将GDP甘露糖转化为GDP岩藻糖，催化反应的酶为GDP甘露糖-4, 6-脱水酶（GDP-mannose-4, 6-dehydratase, GMDS）^[11, 12]^和GDP-4-酮-6-脱氧甘露糖-3, 5异构酶-4-还原酶（GDP-4-keto-6-deoxymannose-3, 5-epimerase-4-reductase, FX）^[13]^；补救合成途径：通过两步反应将L-岩藻糖转化为GDP岩藻糖，催化反应的酶为L-岩藻糖激酶^[14]^和GDP岩藻糖焦磷酸酶^[15]^。哺乳动物细胞仅有中10%GDP岩藻糖由补救合成途径合成，因此从头合成途径在GDP岩藻糖合成中起关键作用^[4]^。

细胞中所有的岩藻糖基化都是由岩藻糖基转移酶（fucosyltransferases, FUT）催化。目前已知的人类岩藻糖基化转移酶有13个^[4]^。蛋白氧岩藻糖基转移酶1、2（protein O-fucosyltransferase, POFUT）定位于内质网腔，其余定位于高尔基体^[4, 16, 17]^。FUT根据功能不同可分为四组，Fut1、Fut2参与α1-2岩藻糖合成，Fut3-7、Fut9-11参与α1-3岩藻糖合成，Fut3、5参与α1-4岩藻糖合成，Fut8参与α1-6岩藻糖合成。Fut1-7、Fut9-11参与末端岩藻糖基化修饰，将岩藻糖基与N-糖或O-糖的末端糖基相连；Fut8参与核心岩藻糖基化修饰，将岩藻糖基与N-糖最内端的N乙酰葡萄糖相连^[18, 19]^（图 1）。

**1:**
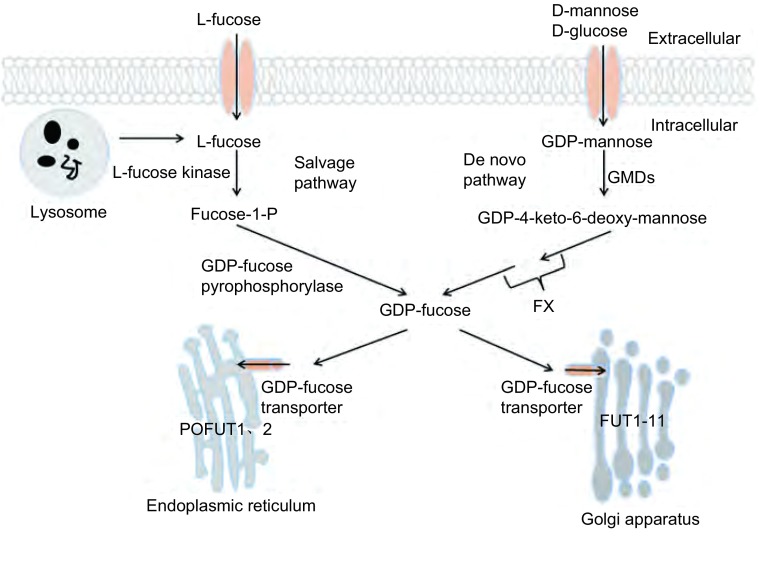
生理条件下岩藻糖基化过程。GDP岩藻糖通过从头合成途径及补救合成途径在胞质内进行合成，然后通过GDP岩藻糖转运体进入内质网或者高尔基体，最终在内质网（POFUT1、2）及高尔基体（FUT1-11）中将岩藻糖转移到受体上。 Fucosylation pathways. GDP-fucose is synthesized in the cytoplasm from GDP-mannose (*de novo* pathway) or L-fucose (salvage pathway). In case it is compounded, GDP-fucose is transported into the ER/Golgi through GDP-fucose transporters. Fucose is transferred from GDP-fucose to acceptors in ER (POFUT1, 2) or Golgi (FUT1-11).

### 1.2 肿瘤中异常岩藻糖基化及其调节

肺癌、结直肠癌、乳腺癌、卵巢癌、肝癌、胰腺癌等多种肿瘤中均存在岩藻糖基化异常^[20, 21]^。GDP岩藻糖合成酶、岩藻糖转运体、岩藻糖基转移酶参与其中，其与肿瘤的分期、预后等密切相关。各种酶在不同肿瘤中的表达情况存在差异；同一种酶在肿瘤的不同病理类型以及不同阶段表达情况也不完全相同。

#### 1.2.1 肿瘤中GDP岩藻糖合成、转运异常

结直肠肿瘤细胞系^[22]^、肝细胞肝癌组织^[23]^中存在FX高表达。与原位结直肠肿瘤细胞系sw480相比，转移性肿瘤细胞系sw620中高表达FX，其与细胞SLea的表达成正相关，并可增强肿瘤-内皮细胞粘附^[22]^。Moriwaki等^[24]^在结肠癌细胞系HCT116中发现GMDS外显子5、6、7缺失从而导致酶活性丧失，细胞岩藻糖基化缺失。同时该研究组在胃绒毛膜癌患者网膜转移组织中也发现了*GMDS*突变（外显子2、3、4缺失）。在肝细胞癌^[23]^及结直肠肿瘤组织^[25]^中存在GDP-L-Fuc Tr高表达。Moriwaki^[23]^的结果表明，GDP-L-Fuc Tr在肝癌细胞岩藻糖基化中发挥关键性作用。

#### 1.2.2 肿瘤中岩藻糖基化修饰异常

##### 1.2.2.1 核心岩藻糖基化异常

Fut8参与核心岩藻糖基化修饰。肺癌^[26]^、乳腺癌^[27]^、肝癌^[28]^、结直肠癌^[29]^、卵巢癌^[30]^、甲状腺癌^[31]^等肿瘤组织中存在Fut8高表达，在胃癌^[32]^组织中Fut8表达下降，这种组织差异性的具体原因目前还不清楚，可能在不同组织中蛋白核心岩藻糖基化的功能不同。

##### 1.2.2.2 末端岩藻糖基化异常

Fut1-7、Fut9-11均参与末端岩藻糖基化修饰，形成特异的lewis血型抗原，如Ⅰ型lewis抗原表位Le^a^、sLe^a^、Le^b^和Ⅱ型lewis抗原表位Le^x^、sLe^x^、Le^y^。其中的α1-2岩藻糖基化由Fut1、Fut2催化，α1-3岩藻糖基化由Fut3-7、Fut9催化，α1-4岩藻糖化由Fut3催化^[27]^。非小细胞肺癌（non-small cell lung cancer, NSCLC）中存在Le^y^、sLe^x^、sLe^a^，三者在不同病理类型中表达存在很大差异，在腺癌中的表达最高，鳞癌和大细胞癌次之，与不表达者相比，表达者具有相对短的生存期^[33]^，乳腺癌中表达Le^x^、sLe^x^、Le^y^，高表达sLe^x^、Le^y^与淋巴结转移、不良预后相关^[34-36]^。结直肠癌中Le^a^、sLe^a^、Le^x^、sLe^x^，表达Le^x^、sLe^x^的患者具有较短的生存期^[37]^。卵巢癌中表达Le^a^、sLe^a^、Le^y^，不同病理类型中存在较大差异，粘液性肿瘤中表达Le^a^、sLe^a^，而浆液性肿瘤中主要表达Le^y[38]^。这些结果表明，末端岩藻糖基化与肿瘤患者预后密切相关；在不同肿瘤组织、同一肿瘤不同病理类型中，末端岩藻糖基化存在较大差异。

## 2 岩藻糖基化与肿瘤增殖、侵袭、转移

肿瘤的增殖、侵袭、转移是复杂的级联反应过程，包括原位肿瘤的增殖，向基底膜、血管侵袭移动，穿入血管，与特定靶器官处血管内皮粘附、穿出，最终在靶器官定植、增殖^[39]^。岩藻糖基化均参与了这一过程。

Ley存在于多种肿瘤组织中，与肿瘤增殖相关，其α1-3岩藻糖基化由Fut4催化^[40-41]^，人表皮鳞癌细胞系A431中过表达Fut4后，其增殖能力增加，S期细胞所占比例提高，进一步研究表明，Fut4过表达通过激活丝裂原活化蛋白激酶（mitogen-activated protein kinase, MAPK）以及磷脂酰肌醇3-激酶（phosphatidylinositol 3-kinase, PI3K）/蛋白激酶B（protein kinase B, PKB/Akt）通路，增加周期蛋白依赖性蛋白激酶（cyclin-dependent protein kinases, CDKs）及细胞周期蛋白（cyclins）表达，降低CDK抑制剂P21、P27表达^[42]^。

整合素家族与细胞生长、增殖、凋亡抵抗和恶性转化密切相关，α3β1整合素参与肿瘤侵袭、转移^[43, 44]^。Fut8敲除的鼠胚胎纤维母细胞α3β1整合素核心岩藻糖基化缺失，导致下游的FAK磷酸化水平降低，细胞侵袭、移动能力下降^[45]^。

表皮-间质转化是肿瘤侵袭、转移的重要起始^[39]^，在此过程中，表皮细胞丢失细胞-细胞粘附特性，增加运动功能及侵袭能力。转化生长因子β（transforming growth factor β, TGF-β）是不同细胞表皮-间质转化的主要诱导因子，介导肿瘤的侵袭转移，结直肠癌细胞系中敲除Fut3和/或Fut6，抑制TGF-β诱导的表皮-间质转化，抑制细胞侵袭能力^[46]^。乳腺癌细胞系中敲除Fut4，表皮标志物E-cadherin表达增加，间质标志物纤连蛋白、波形蛋白、N-cadherin、Snail、Twist、ZEB1表达下降，PI3K/Akt-糖原合成酶激酶（glycogen synthase kinases-3β, GSK3β）信号通路及细胞核因子酉乙蛋白（nuclear factor kappa B, NF-κB）信号通路参与其中^[47]^。

选择素与肿瘤细胞表面sLe^a^、sLe^x^相互作用，促进肿瘤细胞与内皮细胞的粘附，从而促进肿瘤细胞穿出血管^[48, 49]^。过表达FX促进结肠癌细胞与内皮细胞的粘附，而敲除FX降低其与内皮细胞的粘附^[22]^。表达Fut7肺腺癌细胞系A125能够在血管内皮细胞上滚动，而缺乏Fut7转染肺腺癌细胞系A125却不能^[50]^。造血系统细胞HL60，KG1a表达Fut4、7，结肠肿瘤细胞colo205表达Fut3、4、6，前列腺癌细胞MDA PCa2b表达Fut3，Fut3敲除抑制了循环肿瘤细胞在血管内皮细胞上的滚动，而不影响血液系统细胞与内皮细胞的粘附^[51]^，可作为肿瘤治疗的策略之一。

## 3 岩藻糖基化异常与肿瘤免疫逃逸

Moriwaki等^[24]^发现结肠癌细胞系HCT-116细胞*GMDS*突变，导致细胞岩藻糖基化水平下降，与肿瘤形成、发展、转移密切相关。体外和体内实验表明，*GMDS*突变HCT-116细胞能够逃避自然杀伤（natural killer, NK）细胞介导的免疫监视，抵抗肿瘤坏死因子相关凋亡诱导配体（tumor necrosis factor-related apoptosis-inducing ligand, TRAIL）诱导的细胞凋亡。同时，HCT-116细胞对CD95L的刺激也产生抵抗，其可能原因是GMDS突变抑制了Fas相关死亡结构域（Fas-aasociated death domain, FADD）依赖的复合物Ⅱ中Caspase 8的激活^[52]^。人白血病细胞K562中转染Fut7后，NK细胞对高表达sLe^x^细胞的杀伤效能是野生型K562细胞的2.5倍，表明细胞表面sLe^x^的存在促进NK细胞的杀伤活性，而肿瘤细胞因低表达sLe^x^从而逃避NK细胞的杀伤^[53]^。

## 4 岩藻糖基化与表皮生长因子受体

表皮生长因子受体（epidermal growth factor receptor, EGFR）是细胞膜表面的糖蛋白，具有酪氨酸激酶活性，与肿瘤的发生发展密切相关。EGFR具有潜在的12个N糖基化位点^[54]^，N糖基化抑制剂明显降低EGF与EGFR的结合^[55]^，A431肺腺癌细胞系中10个位点存在糖基化，其中7个位点存在岩藻糖基化修饰^[56]^。卵巢癌RMG-I细胞系中转染α1-2岩藻糖转移酶后，细胞增殖能力增强，其通过EGFR发挥作用，Akt、ERK1/2参与其中^[57]^。FUT8敲除的小鼠胚胎纤维母细胞，EGFR岩藻糖基化及磷酸化水平明显下降，其下游信号分子JNK、ERK1/2磷酸化水平也受到抑制，同时亲合力测试发现，核心岩藻糖基化的EGFR与EGF的亲和力更高^[58]^，因此，岩藻糖基转移酶通过对EGFR的活性产生影响，从而导致肿瘤的发生发展。

## 5 岩藻糖基化与肺癌

复旦大学的研究^[59]^显示，13例转移性肺癌患者中12例存在E-cadherin核心岩藻糖基化，而5例原位组织中E-cadherin均无核心岩藻糖基化，说明E-cadherin核心岩藻糖基化参与肿瘤的转移，进一步研究表明核心岩藻糖基化可能破坏了E-cadherin N糖的三维结构，从而影响其功能。

台湾大学的研究^[60]^表明，肺腺癌细胞系CL1-5与CL1-0相比具有高侵袭能力，同时CL1-5具有更高的岩藻糖基化，其Fut8表达是CL1-0的22.5倍，说明岩藻糖基化与肿瘤的侵袭力相关。同时，肺癌细胞系A549中过表达Fut4/6导致EGFR二聚化、磷酸化水平下降，而CL1-0及A549细胞系中过表达Fut8对EGFR活性却无影响，CL1-5细胞系中敲除Fut8后EGFR二聚化、磷酸化下降，提示过表达Fut4/6抑制了肺癌细胞中EGFR二聚化、磷酸化，同时表明FUT8在维持肺癌细胞EGFR二聚化、磷酸化中起关键作用。其后的研究中，该课题组^[26]^发现，140例Ⅰ期-Ⅳ期的NSCLC患者（86例肺腺癌，46例肺鳞癌及8例肺大细胞癌）中均存在Fut8表达，在肺腺癌中Fut8表达水平最高，Fut8表达水平与转移复发相关，并且Fut8高表达者具有更短的无病生存期和总生存期，同时体外研究表明，过表达Fut8增加肺癌细胞系的增殖侵袭和转移能力。其后，日本北海道大学和大阪大学的研究人员^[61]^发现，在Ⅰ期及可治愈的外科切除的129例NSCLC患者中，高表达Fut8与不良预后相关。这些结果表明，核心岩藻糖基化与肺癌增殖、侵袭、转移以及疾病预后密切相关。靶向肿瘤中Fut8可能成为治疗肺癌的新策略。

另外，在含表皮生长因子（epidermal growth factor, EGF）的培养条件下，沉默A549细胞中Fut8后，EGFR及MAPK磷酸化水平下降，其对吉非替尼的敏感性明显下降，表明Fut8下调可降低野生型EGFR对酪氨酸激酶抑制剂（tyrosine kinase inhibitor, TKI）的敏感性^[62]^。同时这也可能是野生型EGFR高表达患者对吉非替尼有效的原因，其是否可以作为野生型患者EGFR-TKI疗效的预测因子有待进一步研究。

然而，其他岩藻糖基转移酶及GDP岩藻糖合成酶（GMDS和FX）在肺癌中的表达及在肿瘤发生发展中的作用目前还未知。

## 6 总结

肿瘤细胞岩藻糖基化异常参与细胞表面受体EGFR、TGF-βR的激活，影响整合素、选择素、凋亡信号通路的功能，并最终参与肿瘤增殖、凋亡、侵袭、转移及药物敏感性。大多数肿瘤中都存在岩藻糖基化异常，因此针对岩藻糖基化异常治疗肿瘤将成为可能。然而，在不同肿瘤类型、同一肿瘤不同病理类型以及同一肿瘤的不同发展阶段，其岩藻糖基化都有可能不同。其次，在不同肿瘤类型中，岩藻糖基化过程中酶的作用也可能不同，结直肠细胞系sw620中高表达FX可能增加了肿瘤细胞的转移能力，HCT116细胞中GMDS功能缺失促进了肿瘤细胞的免疫逃逸。这给肿瘤中岩藻糖基化的研究带来了挑战。目前，对岩藻糖基化的研究还处于基础阶段，我们的首要工作是弄清各种酶在不同肿瘤中的作用及其机制。相信，岩藻糖基化的研究会有一个美好的前景，针对肿瘤中异常岩藻糖基化的治疗会给肿瘤患者带来曙光。

## References

[1] Agard NJ, Bertozzi CR (2009). Chemical approaches to perturb, profile, and perceive glycans. Acc Chem Res.

[2] Gloster TM, Vocadlo DJ (2012). Developing inhibitors of glycan processing enzymes as tools for enabling glycobiology. Nat Chem Biol.

[3] Kiessling LL, Splain RA (2010). Chemical approaches to glycobiology. Annu Rev Biochem.

[4] Becker DJ, Lowe JB (2003). Fucose: biosynthesis and biological function in mammals. Glycobiology.

[5] van de Vijver E, Maddalena A, Sanal O (2012). Hematologically important mutations: Leukocyte adhesion deficiency. Blood Cells Mol Dis.

[6] Baumann H, Nudelman E, Watanabe K (1979). Neutral fucolipids and fucogangliosides of rat hepatoma HTC and H35 cells, rat liver, and hepatocytes. Cancer Res.

[7] Aoyagi Y, Isemura M, Suzuki Y (1985). Fucosylated alpha-fetoprotein as marker of early hepatocellular carcinoma. Lancet.

[8] Aoyagi Y, Isemura M, Suzuki Y (1986). Change in fucosylation of alpha-fetoprotein on malignant transformation of liver cells. Lancet.

[9] Lübke T, Marquardt T, Etzioni A (2001). Complementation cloning identifies CDG-IIc, a new type of congenital disorders of glycosylation, as a GDP-fucose transporter deficiency. Nat Genet.

[10] Lühn K, Wild MK, Eckhardt M (2001). The gene defective in leukocyte adhesion deficiency Ⅱ encodes a putative GDP-fucose transporter. Nat Genet.

[11] Sullivan FX, Kumar R, Kriz R (1998). Molecular cloning of human GDP-mannose 4, 6-dehydratase and reconstitution of GDP-fucose biosynthesis *in vitro*. J Biol Chem.

[12] Tonetti M, Sturla L, Bisso A (1996). Synthesis of GDP-L-fucose by the human FX protein. J Biol Chem.

[13] Smith PL, Myers JT, Rogers CE (2002). Conditional control of selectin ligand expression and global fucosylation events in mice with a targeted mutation at the FX locus. J Cell Biol.

[14] Park SH, Pastuszak I, Drake R (1998). Purification to apparent homogeneity and properties of pig kidney L-fucose kinase. J Biol Chem.

[15] Pastuszak I, Ketchum C, Hermanson G (1998). GDP-L-fucose pyrophosphorylase. Purification, cDNA cloning, and properties of the enzyme. J Biol Chem.

[16] Luo Y, Koles K, Vorndam W (2006). Protein O-fucosyltransferase 2 adds O-fucose to thrombospondin type 1 repeats. J Biol Chem.

[17] Luo Y, Haltiwanger RS (2005). O-fucosylation of Notch occurs in the endoplasmic reticulum. J Biol Chem.

[18] Ma B, Simala-Grant JL, Taylor DE (2006). Fucosylation in prokaryotes and eukaryotes. Glycobiology.

[19] Mollicone R, Moore SE, Bovin N Activity, splice variants, conserved peptide motifs, and phylogeny of two new alpha1, 3-fucosyltransferase families (FUT10 and FUT11). J Biol Chem.

[20] Miyoshi E, Moriwaki K, Nakagawa T (2008). Biological function of fucosylation in cancer biology. J Biochem.

[21] Christiansen MN, Chik J, Lee L (2014). Cell surface protein glycosylation in cancer. Proteomics.

[22] Zipin A, Israeli-Amit M, Meshel T (2004). Tumor-microenvironment interactions: the fucose-generating FX enzyme controls adhesive properties of colorectal cancer cells. Cancer Res.

[23] Moriwaki K, Noda K, Nakagawa T (2007). A high expression of GDP-fucose transporter in hepatocellular carcinoma is a key factor for increases in fucosylation. Glycobiology.

[24] Moriwaki K, Noda K, Furukawa Y (2009). Deficiency of GMDS leads to escape from NK cell-mediated tumor surveillance through modulation of TRAIL signaling. Gastroenterology.

[25] Villar-Portela S, Muinelo-Romay L, Cuevas E (2013). FX enzyme and GDP-L-Fuc transporter expression in colorectal cancer. Histopathology.

[26] Chen CY, Jan YH, Juan YH (2013). Fucosyltransferase 8 as a functional regulator of non-small cell lung cancer. Proc Natl Acad Sci U S A.

[27] Potapenko IO, Haakensen VD, Lüders T (2010). Glycan gene expression signatures in normal and malignant breast tissue; possible role in diagnosis and progression. Mol Oncol.

[28] Noda K, Miyoshi E, Uozumi N (1998). Gene expression of alpha1-6 fucosyltransferase in human hepatoma tissues: a possible implication for increased fucosylation of alpha-fetoprotein. Hepatology.

[29] Muinelo-Romay L, Vazquez-Martín C, Villar-Portela S (2008). Expression and enzyme activity of alpha(1, 6)fucosyltransferase in human colorectal cancer. Int J Cancer.

[30] Abbott KL, Nairn AV, Hall EM (2008). Focused glycomic analysis of the N-linked glycan biosynthetic pathway in ovarian cancer. Proteomics.

[31] Ito Y, Miyauchi A, Yoshida H (2003). Expression of alpha1, 6-fucosyltransferase (FUT8) in papillary carcinoma of the thyroid: its linkage to biological aggressiveness and anaplastic transformation. Cancer Lett.

[32] Zhao YP, Xu XY, Fang M (2014). Decreased core-fucosylation contributes to malignancy in gastric cancer. PLoS One.

[33] Ogawa J, Sano A, Inoue H (1995). Expression of Lewis-related antigen and prognosis in stage Ⅰ non-small cell lung cancer. Ann Thorac Surg.

[34] Listinsky JJ, Siegal GP, Listinsky CM (2011). The emerging importance of alpha-L-fucose in human breast cancer: a review. Am J Transl Res.

[35] Ura Y, Dion AS, Williams CJ (1992). Quantitative dot blot analyses of blood-group-related antigens in paired normal and malignant human breast tissues. Int J Cancer.

[36] Madjd Z, Parsons T, Watson NF (2005). High expression of Lewis y/b antigens is associated with decreased survival in lymph node negative breast carcinomas. Breast Cancer Res.

[37] Nakagoe T, Fukushima K, Nanashima A (2000). Expression of Lewis(a), sialyl Lewis(a), Lewis(x) and sialyl Lewis(x) antigens as prognostic factors in patients with colorectal cancer. Can J Gastroenterol.

[38] Federici MF, Kudryashov V, Saigo PE (1999). Selection of carbohydrate antigens in human epithelial ovarian cancers as targets for immunotherapy: serous and mucinous tumors exhibit distinctive patterns of expression. Int J Cancer.

[39] Spano D, Heck C, De Antonellis P (2012). Molecular networks that regulate cancer metastasis. Semin Cancer Biol.

[40] Taniguchi A, Suga R, Matsumoto K (2000). Expression and transcriptional regulation of the human alpha1, 3-fucosyltransferase 4 (*FUT4*) gene in myeloid and colon adenocarcinoma cell lines. Biochem Biophys Res Commun.

[41] Escrevente C, Machado E, Brito C (2006). Different expression levels of alpha3/4 fucosyltransferases and Lewis determinants in ovarian carcinoma tissues and cell lines. Int J Oncol.

[42] Yang XS, Liu S, Liu YJ (2010). Overexpression of fucosyltransferase Ⅳ promotes A431 cell proliferation through MAPK and PI3K_Akt pathway. J Cell Physiol.

[43] Seguin L, Desgrosellier JS, Weis SM (2015). Integrins and cancer: regulators of cancer stemness, metastasis, and drug resistance. Trends Cell Biol.

[44] Melchiori A, Mortarini R, Carlone S (1995). The alpha 3 beta 1 integrin is involved in melanoma cell migration and invasion. Exp Cell Res.

[45] Zhao Y, Itoh S, Wang X (2006). Deletion of core fucosylation on α3β1 integrin down-regulates its functions. J Biol Chem.

[46] Hirakawa M, Takimoto R, Tamura F (2014). Fucosylated TGF-β receptors transduces a signal for epithelial-mesenchymal transition in colorectal cancer cells. Br J Cancer.

[47] Yang X, Liu S, Yan Q (2013). Role of fucosyltransferase Ⅳ in epithelial-mesenchymal transition in breast cancer cells. Cell Death Dis.

[48] McEver RP (1997). Selectin-carbohydrate interactions during inflammation and metastasis. Glycoconj J.

[49] Sass PM (1998). The involvement of selectins in cell adhesion, tumor progression, and metastasis. Cancer Invest.

[50] Friederichs J, Zeller Y, Hafezi-Moghadam A (2000). The CD24/P-selectin binding pathway initiates lung arrest of human a125 adenocarcinoma cells. Cancer Res.

[51] Yin X, Rana K, Ponmudi V (2010). Knockdown of fucosyltransferase Ⅲ disrupts the adhesion of circulating cancer cells to E-selectin without affecting hematopoietic cell adhesion. Carbohydr Res.

[52] Moriwaki K, Shinzaki S, Miyoshi E (2011). GDP-mannose-4, 6-dehydratase (GMDS) deficiency renders colon cancer cells resistant to tumor necrosis factorrelated apoptosis-inducing ligand (TRAIL) receptor-and CD95-mediated apoptosis by inhibiting complex Ⅱ formation. J Biol Chem.

[53] Higai K, Ichikawa A, Matsumoto K (2006). Binding of sialyl Lewis X antigen to lectin-like receptors on NK cells induces cytotoxicity and tyrosine phosphorylation of a 17-kDa protein. Biochim Biophys Acta.

[54] Ullrich A, Coussens L, Hayflick JS (1984). Human epidermal growth factor receptor cDNA sequence and aberrant expression of the amplified gene in A431 epidermoid carcinoma cells. Nature.

[55] Soderquist AM, Carpenter G (1984). Glycosylation of the epidermal growth factor receptor in A-431 cells. The contribution of carbohydrate to receptor function. J Biol Chem.

[56] Wu SL, Taylor AD, Lu Q (2013). Identification of potential glycan cancer markers with sialic acid attached to sialic acid and up-regulated fucosylated galactose structures in epidermal growth factor receptor secreted from A431 cell line. Mol Cell Proteomics.

[57] Liu JJ, Lin B, Hao YY (2010). Lewis(y) antigen stimulates the growth of ovarian cancer cells via regulation of the epidermal growth factor receptor pathway. Oncol Rep.

[58] Wang X, Gu J, Ihara H (2006). Core fucosylation regulates epidermal growth factor receptor-mediated intracellular signaling. J Biol Chem.

[59] Geng F, Shi BZ, Yuan YF (2004). The expression of core fucosylated E-cadherin in cancer cells and lung cancer patients: prognostic implications. Cell Res.

[60] Liu YC, Yen HY, Chen CY (2011). Sialylation and fucosylation of epidermal growth factor receptor suppress its dimerization and activation in lung cancer cells. Proc Natl Acad Sci U S A.

[61] Honma R, Kinoshita I, Miyoshi E (2015). Expression of fucosyltransferase 8 is associated with an unfavorable clinical outcome in non-small cell lung cancers. Oncology.

[62] Matsumoto K, Yokote H, Arao T (2008). N-Glycan fucosylation of epidermal growth factor receptor modulates receptor activity and sensitivity to epidermal growth factor receptor tyrosine kinase inhibitor. Cancer Sci.

